# Effectively supporting widening participation learners in medical education through a capability approach lens

**DOI:** 10.5116/ijme.66d8.1a0f

**Published:** 2024-09-20

**Authors:** Pirashanthie Vivekananda-Schmidt, John Sandars, Lopa Husain, Peter Leadbetter, Michelle Marshall

**Affiliations:** 1 School of Medicine and Population Health, University of Sheffield, Beech Hill Road, Sheffield, UK; 2 Edge Hill University, St Helens Road, Ormskirk, Lancashire, UK

**Keywords:** Widening participation, medical education, holistic dimensions, capability approach

## Introduction

There have been increasing numbers of widening participation (WP) learners in medical education and this has been accompanied by an increased challenge of supporting these learners.  In this editorial, we advocate for the implementation of WP learner support that has a flourishing perspective, where the focus is on enhancing how each learner can achieve their own psycho-social and academic potential. We propose that medical educators adopt a capability approach lens to understand the flourishing of each WP learner, including the enabling and constraining factors, and to use this understanding to inform holistic support.

### The challenge of increasing widening access and providing support for learners in medical education

Diversifying the future medical workforce has been proposed as an urgent key action to reduce population health inequalities in many regions of the world, including Europe, Australia and New Zealand, and US and Canada, with the aim of improving the health of individuals and their communities.^1^^-^^4^ A key response to this proposal, has been numerous educational policy initiatives to increase the access of a diverse range of the learners to medicine, especially from specific lower socio-economic, cultural and academic backgrounds.^5^^,^^6^An example of this work in the UK is the Medical School Council Selection Alliance.^4^

Despite these initiatives, there have been increasing concerns by educators that the increased diversity of widening access (WA) learners in medical education has been associated with a greater increase in the challenge of supporting this group of learners.^7^ For example, Harrison and colleagues^8^  found that access to higher education alone is insufficient if these learners do not have an opportunity to fully benefit from their participation in the range of university experiences available to them. This highlights the important difference between the use of the terms WA and WP.  Our particular interest has been WP learners in medical education since we have a responsibility for providing support for this group of learners and we frequently encounter how they struggle to achieve their potential, both in psycho-social development and academic achievement.

The medical education experiences of WP learners is often limited by their socio-economic, cultural and educational resources that prepare them for study at medical school, including insufficient access to medical career and academic role-models, mentors and advisors.^9^ It has also become increasingly recognised that the support of these learners is often influenced by biases from peers and faculty staff, especially related to low expectations about their psycho-social development and academic potential.^10^   This low expectation can create a deficit perception of the learner, with the consequence that the focus of support can become limited to resilience training, academic skills preparation and remediation initiatives without consideration of how to support the learner to more holistically flourish as both a person and future doctor.^11^

### The importance of flourishing as a perspective for medical education

We advocate for reframing our understanding of the WP learner to inform our approach for holistically providing support for these learners.  In the wider field of higher education there has been a growing recognition of this need for a more holistic approach that has a focus on flourishing^12^ and increasingly there has been a similar recognition of a flourishing perspective for medical education.^13^ An important aspect of this holistic approach to support is the investment by educators into the flourishing of each individual learner so that they can achieve their psycho-social and academic potential.^14^

Aristotle^15^ explains that flourishing is not to be equated with hedonistic pleasure and satisfaction. Instead, to flourish, individuals should engage in activities driven by moral and intellectual virtues. This engagement can lead to the actualisation of the potential of each individual.  Flourishing can be considered to be a lifelong journey to achieve the best of oneself across several holistic dimensions, including psychosocial wellbeing, development of an integrated personal and professional identity, and academic achievement.^16^

Previous research on flourishing in medical education has mainly focused on the psycho-social dimensions of wellbeing, with the use of established measures of wellbeing, satisfaction of work-life balance and quality of life to inform the support of all learners.^17^ However, there has been a call for further understanding of more holistic dimensions in medical education, especially for the support of WP learners, that recognises the importance of meeting the wide diversity of individual needs of learners with specific characteristics such as race, ethnicity, gender, sexual orientation, socio-economic status, age and religious beliefs.^13^

### Understanding flourishing in education through a capability approach lens

Sen’s capability approach can offer a useful theoretical and practical lens for understanding flourishing in education. It advocates that everyone has a range of individual needs that are important for their life, such as having shelter or feeling respected, and that an individual seeks to achieve their own unique range of valued outcomes that meets their needs.^18^ The achievement of the valued outcomes determines a flourishing life.  An important aspect of the capability approach is the agency of an individual, initially by having the opportunity to make choices about their valued outcomes, and then the opportunity to achieve their chosen valued outcomes.^18^

These opportunities can be enabled or constrained by a wide variety of internal and external influences, which are called conversion factors. Examples of such internal factors include the unique individual characteristics, such as previous life experiences, and external factors, such as the environment in which the individual is living and working.^18^

### Using a capability approach lens for informing the support of WP learners

In the wider field of education, the capability approach lens has also offered a useful theoretical and practical lens through which to view and understand how WP learners can be supported to flourish.^16^ We have recently proposed that this lens is applied to medical education.^19^ Although we have not identified any previous studies, we have experience in using this lens to understand the use of point of care information systems.^20^ We obtained useful insights into the valued outcomes of learners and the barriers to achieving these outcomes. Our current research with WP learners has identified that they were often unable to choose and achieve full participation in the broad range of university experiences to enhance their flourishing, such as student societies and electives. In addition, we have also obtained a further understanding of the conversion factors that limit their flourishing, such as a lack of belongingness and wellbeing services that were not responsive to their specific needs. This has important implications for informing appropriate approaches for providing support, including facilitating peer relationships and wellbeing services.

A capability approach lens could also inform curriculum implementation to improve flourishing and the learning experiences of WP learners in medical education by empowering all learners to make choices about their education so that it meets their needs and valued outcomes.^21^  Learners can be enabled to have more agency over their learning (within the constraints of the prescribed curriculum of core content) by giving them increased opportunities to make choices on topics and resources of individual interest, such as during clinical placements or electives.  For example, learners can be encouraged to explore areas of interest, such as holistic pain management during palliative care placements or developing increased understanding of clinical academic careers through student selected electives.

## Conclusions

The increasing numbers of WP learners in medical education has been accompanied by an increased challenge for providing support for these learners. Reframing the support of the diversity of WP learners to a more holistic focus on enhancing flourishing is essential for supporting each individual to achieve their own psycho-social and academic potential. The capability approach can offer a useful theoretical and practical lens for understanding each WP learner in medical education and for informing holistic support for enhancing flourishing and potential.

### Conflict of Interest

The authors declare that they have no conflict of interest.
